# Supplementary material for: Lipid metabolism adaptations are reduced in human compared to murine Schwann cells following injury
Source: Nat Commun. 2020 May 1;11:2123. doi: 10.1038/s41467-020-15915-4 (PMC7195462; doi:10.1038/s41467-020-15915-4)
Supplement: Supplementary file 1 — Supplementary Information [file 41467_2020_15915_MOESM1_ESM.pdf]

Supplementary Information

Lipid metabolism adaptations are reduced in human compared to murine Schwann cells following injury

Meyer zu Reckendorf et al.

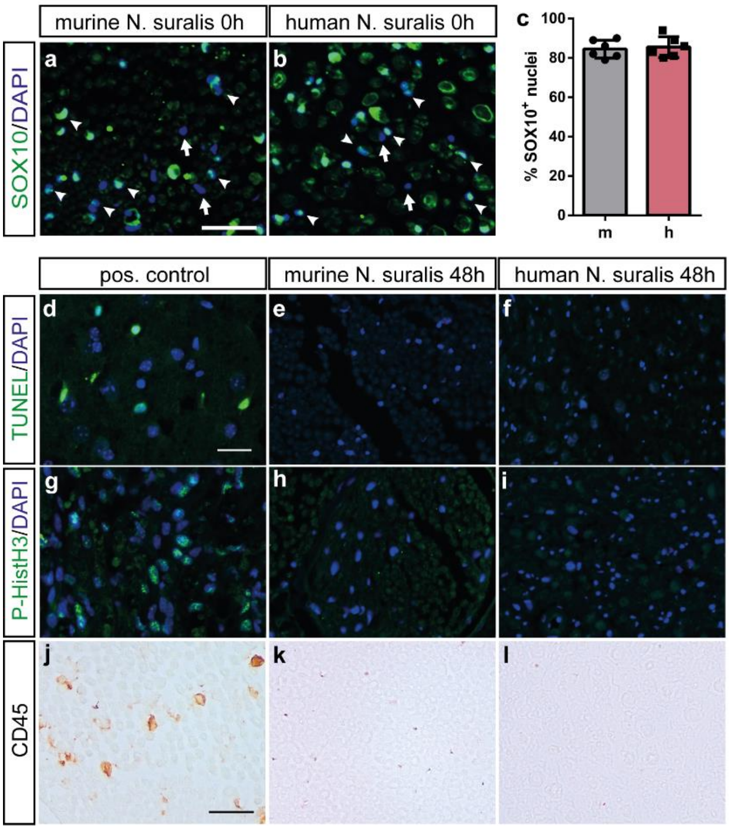

Supplementary Figure 1: SC numbers, apoptosis, proliferation and inflammation in injured nerves

(**a-c**) Uninjured murine (a) and human (b) nerve explants were stained for SOX10 (green) and DAPI (blue). Quantification of $SOX10^+/DAPI^+$ nuclei relative to all $DAPI^+$ nuclei (c) in murine (m) and human (h) nerve explants. Each dot represents one murine or human sample analysed (n = 6 biological replicates for murine and human tissue). Bars show mean with SD.

(**d-f**) TUNEL assay labeling apoptotic cells in murine (e) and human (f) nerve explants 48 h after injury. As positive control, a brain section of a mouse with a traumatic brain injury was used (d).

(**g-i**) P-Histone H3 staining marking proliferating cells in nerve explants 48 h post injury (h, i). As a positive control, an *in vivo* injured nerve 3 days after injury was used (g).

(**j-l**) CD45 staining in nerve explants 48 h after injury (k, l). As a positive control, an *in vivo* injured nerve 3 days post injury was used (j).

For each staining (SOX10, TUNEL, P-Histone H3, CD45) six biological replicates for human and murine tissue were stained. Scale in (a) applies for (a-b). Scale in (d) applies for (d-i). Scale in (j) applies for (j-l). All scales represent 25 µm. Source data for (c) are provided as a Source Data file.

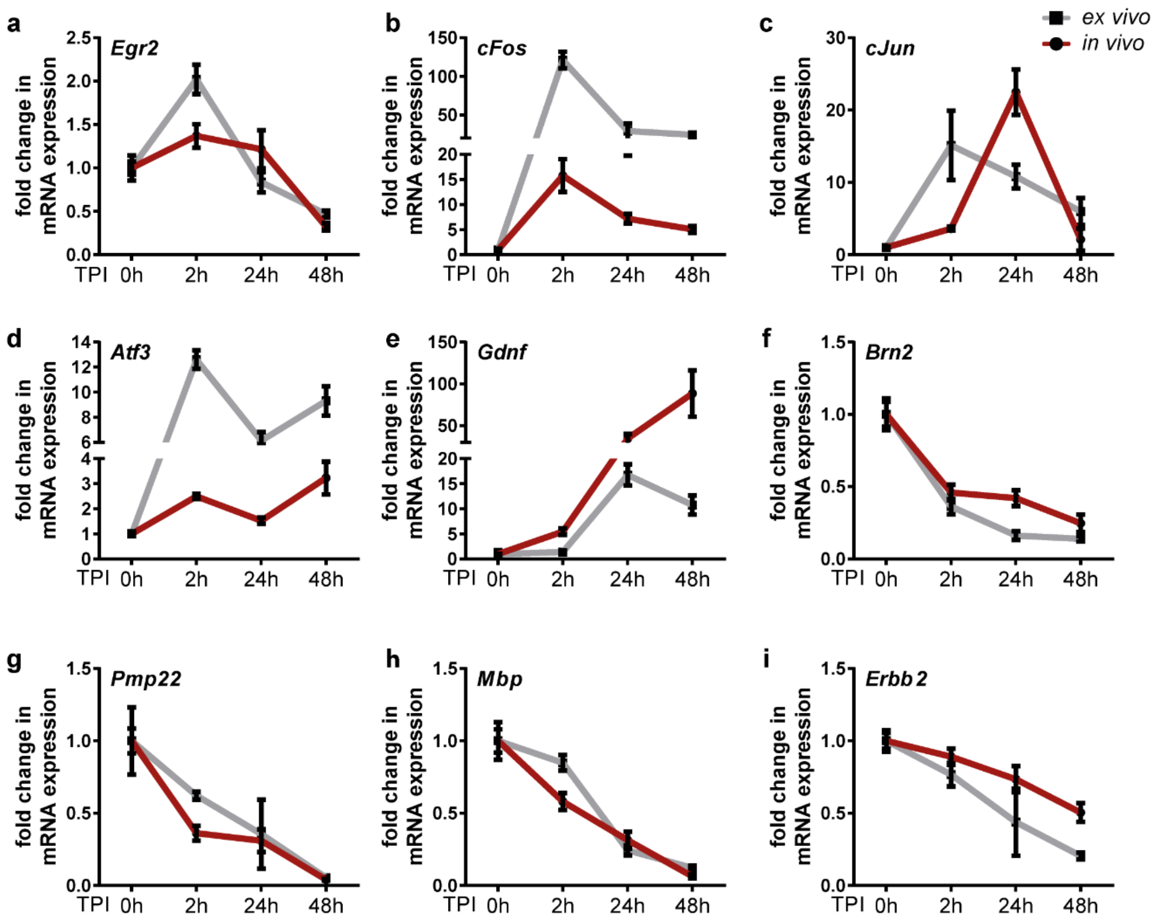

Supplementary Figure 2: Gene expression in injured nerves *in vivo* vs. *ex vivo*

(**a-e**) qPCR analysis of genes typically induced in SCs after injury. Gene expression was analyzed in sciatic nerves injured in living mice (*in vivo*; red lines) or in the *ex vivo* injury model (grey lines).

(**f-i**) qPCR analysis of genes that are highly expressed in differentiated and downregulated in repair SCs. Sciatic nerves were used for both, *ex* and *in vivo* injury. Expression at 0 h was set to one and the fold change was calculated for the time points post injury (TPI).

Graphs show mean with SEM. n at 0 h, 2 h, 24 h and 48 h was 8, 2, 3 and 3 biological replicates for *in vivo* nerves and 4, 5, 4 and 5 biological replicates for *ex vivo* nerves. Source data are provided as a Source Data file.

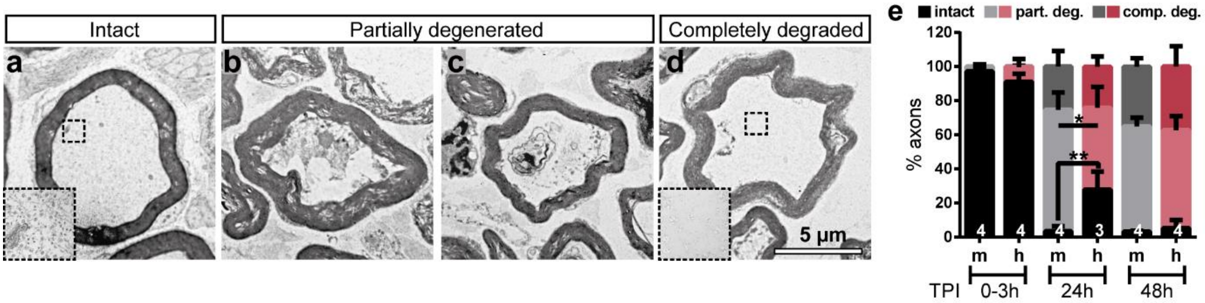

Supplementary Figure 3: Axonal degeneration in injured nerve explants

(**a-d**) Representative images for intact (a), partially degenerated (b, c) or completely degraded axons in electron microscopical images. Insert in (a) and (d) show higher magnifications of the axonal compartment, where tubulin structures were visible in intact but not degraded axons.

(**e**) Quantification of intact, partially degenerated or completely degraded axons at different time points in murine (m) or human (h) nerves. The numbers in the graph indicate the number of independent biological replicates analysed. Bars show mean with SD. Two-sided T-test was used to calculate statistical significance (*P < 0.05, **P < 0.005, ***P < 0.001). P = 0.04 for 24 h partially degenerated, P = 0.0062 for 24 h intact. Source data are provided as a Source Data file.

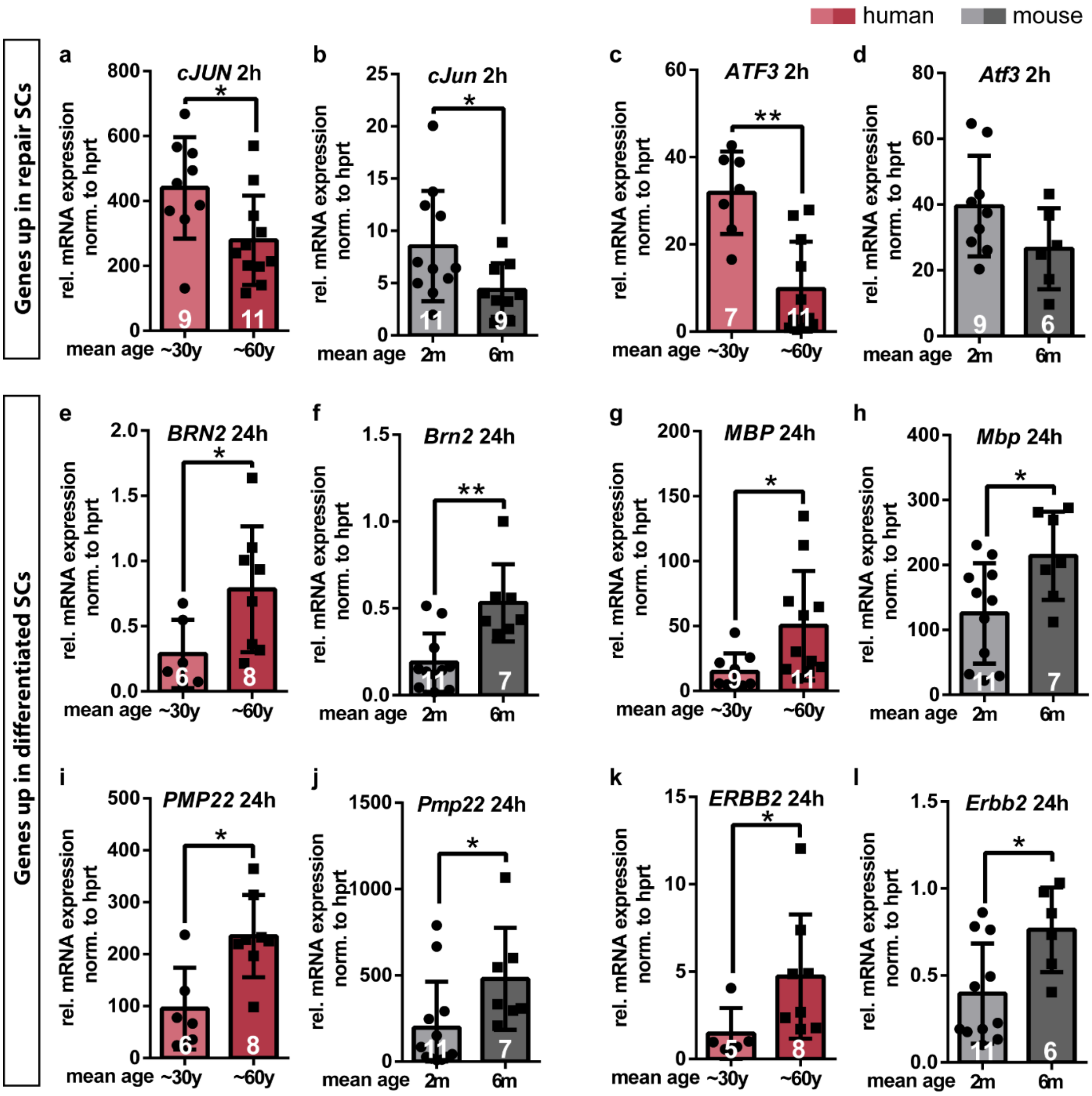

Supplementary Figure 4: Correlation of mRNA abundance after injury with age

(**a-d**) Nerves from mice and patients were separated into two age groups and qPCR analysis was performed for genes highly abundant in repair SCs. For human nerves, all patients up to 50 years of age (mean age ~30 years) were grouped and all patients at least 60 years of age (mean age ~60 years) were placed in the other group. For murine nerves, the “young” group comprised mice with an age of 2 months and mice in the “old” group were 6 months of age. P = 0.0381, 0.0309, 0.0012 for (a), (b), (c) respectively.

(**e-l**) qPCR analysis of genes that are typically expressed at low levels in SCs 24 h after injury. P = 0.0293, 0.0041, 0.0159, 0.0441, 0.0293, 0.0163, 0.0186, 0.0365 for (e), (f), (g), (h), (i), (j), (k), (l), (m) respectively.

Each dot represents a single mouse or human sample. Numbers in the bars indicate the exact n numbers of biological replicates. All error bars show mean with SD. Two-sided Mann-Whitney test was used to calculate statistical significance (*P < 0.05, **P < 0.005, ***P < 0.001). Source data are provided as a Source Data file.

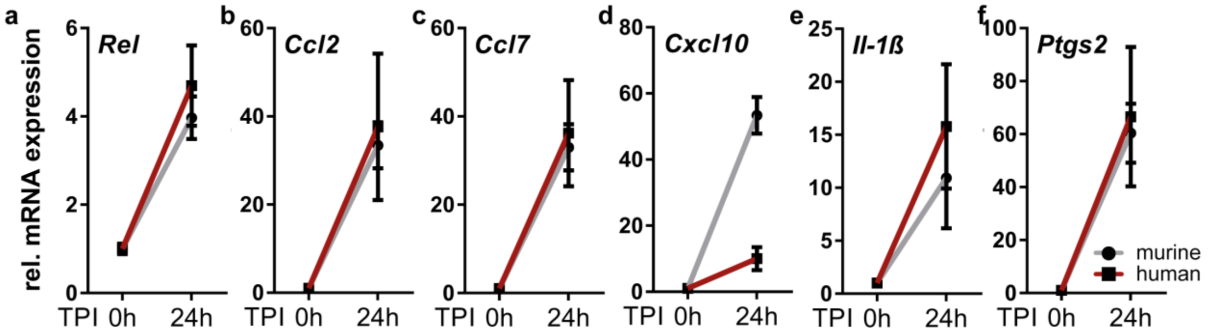

Supplementary Figure 5: Induction of inflammation-related genes in injured nerve explants

(**a-f**) qPCR analysis of inflammation-related genes in injured murine (grey lines) or human (red lines) nerve explants. Expression at 0 h was set to one and the fold change was calculated for the 24 h time point post injury (TPI). Graphs show mean with SEM. n = 6 for each time point for human and murine explants. Source data are provided as a Source Data file.

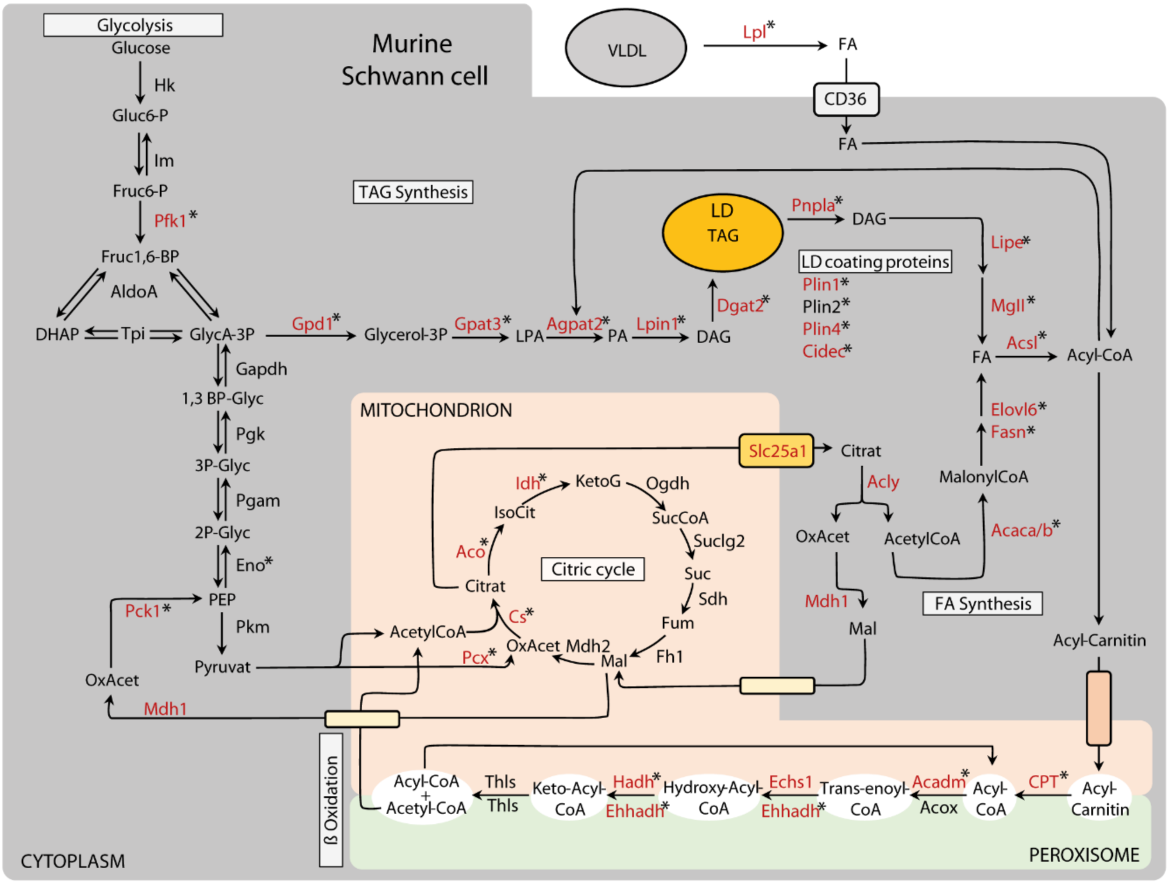

Supplementary Figure 6: Overview of injury induced metabolic changes in SCs

Nerve injury induces SC reprograming, which is accompanied by numerous metabolic changes in murine SCs. The illustration shows an overview of some of the altered metabolic processes observed by genome-wide transcriptomics in cells. Gene expression of enzymes highlighted in red were down-regulated at least two-fold in the microarray analysis of murine nerves 24 h after injury. Asterisks mark genes that have previously been reported to be regulated by PPARg activity.

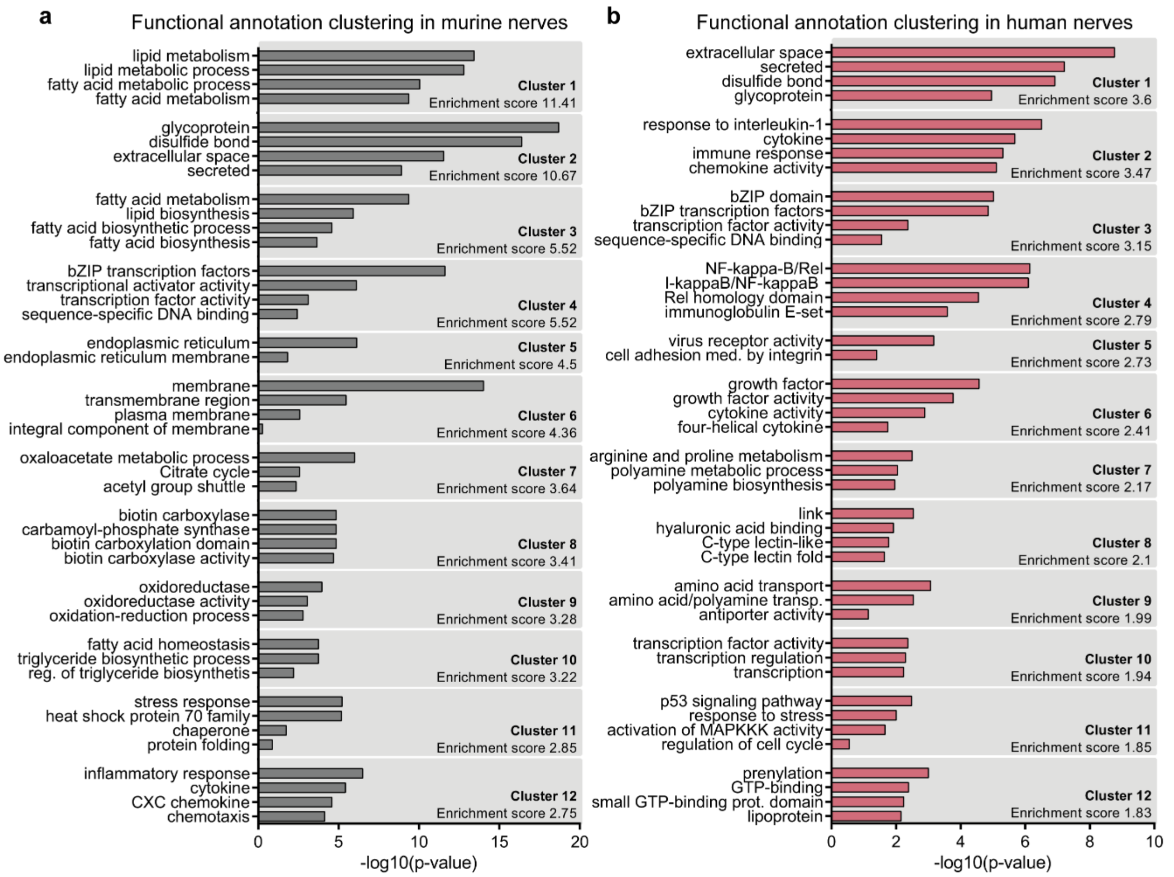

Supplementary Figure 7: Functional annotation clustering of differentially regulated genes 24 h after injury

(**a, b**) The 12 most enriched functional clusters comprising differentially regulated genes in murine (a) and human (b) 24 h after nerve injury. Genes more than two-fold up- or downregulated were included in this analysis. Several functional annotations associated directly (clusters 1, 3, 10) or indirectly to lipid metabolism were among the top enriched clusters in murine nerves, while absent in human nerves. P values were calculated by the online DAVID platform (see Supplementary Methods), which uses the Fisher's Exact test.

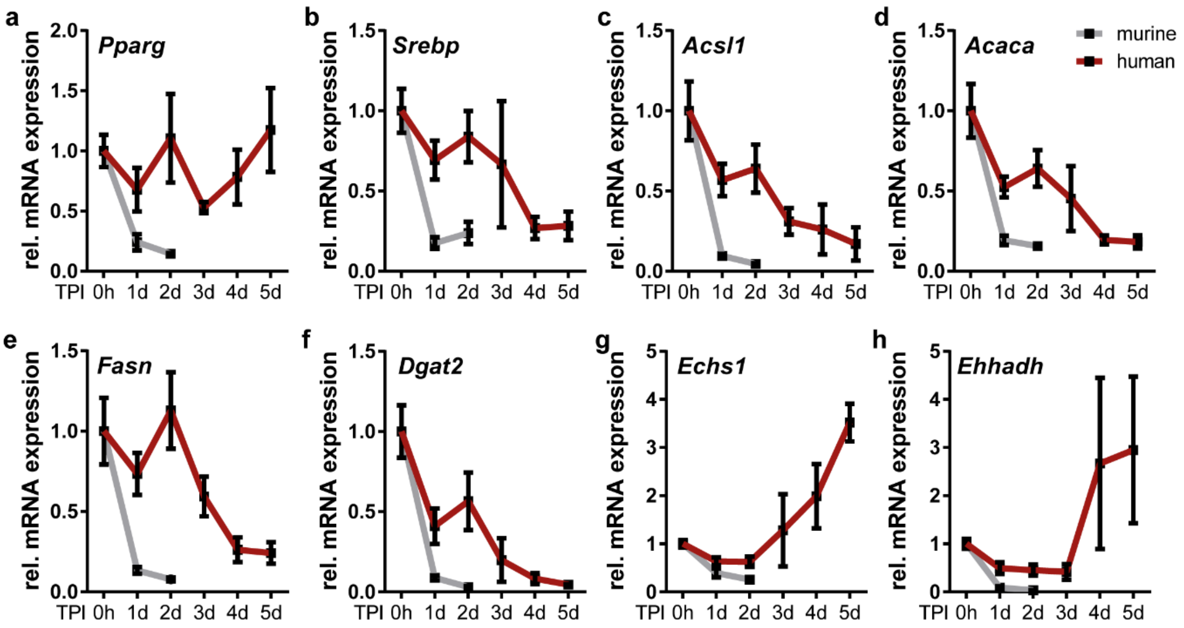

Supplementary Figure 8: Lipogenic gene expression is delayed in injured nerve explants

(**a-h**) qPCR analysis of lipogenic gene expression in injured murine (grey lines) or human (red lines) nerve explants. Expression at 0 h was set to one and the fold change was calculated for the other time point post injury (TPI). Human nerves were incubated for up to 5 days (5d). Murine data were the same as depicted in Fig. 4. Error bars show SEM. Biological replicates human nerves: n = 15, 15, 12, 3, 3, 3 at 0 h, 1 d, 2 d, 3 d, 4 d, 5 d respectively for (a-f) and n = 7, 7, 7, 3, 3, 3 at 0 h, 1 d, 2 d, 3 d, 4 d, 5 d respectively for (g, h). Biological replicates murine nerves: n = 10, 10 and 4 for 0 h, 1 d and 2 d respectively for (a-h).

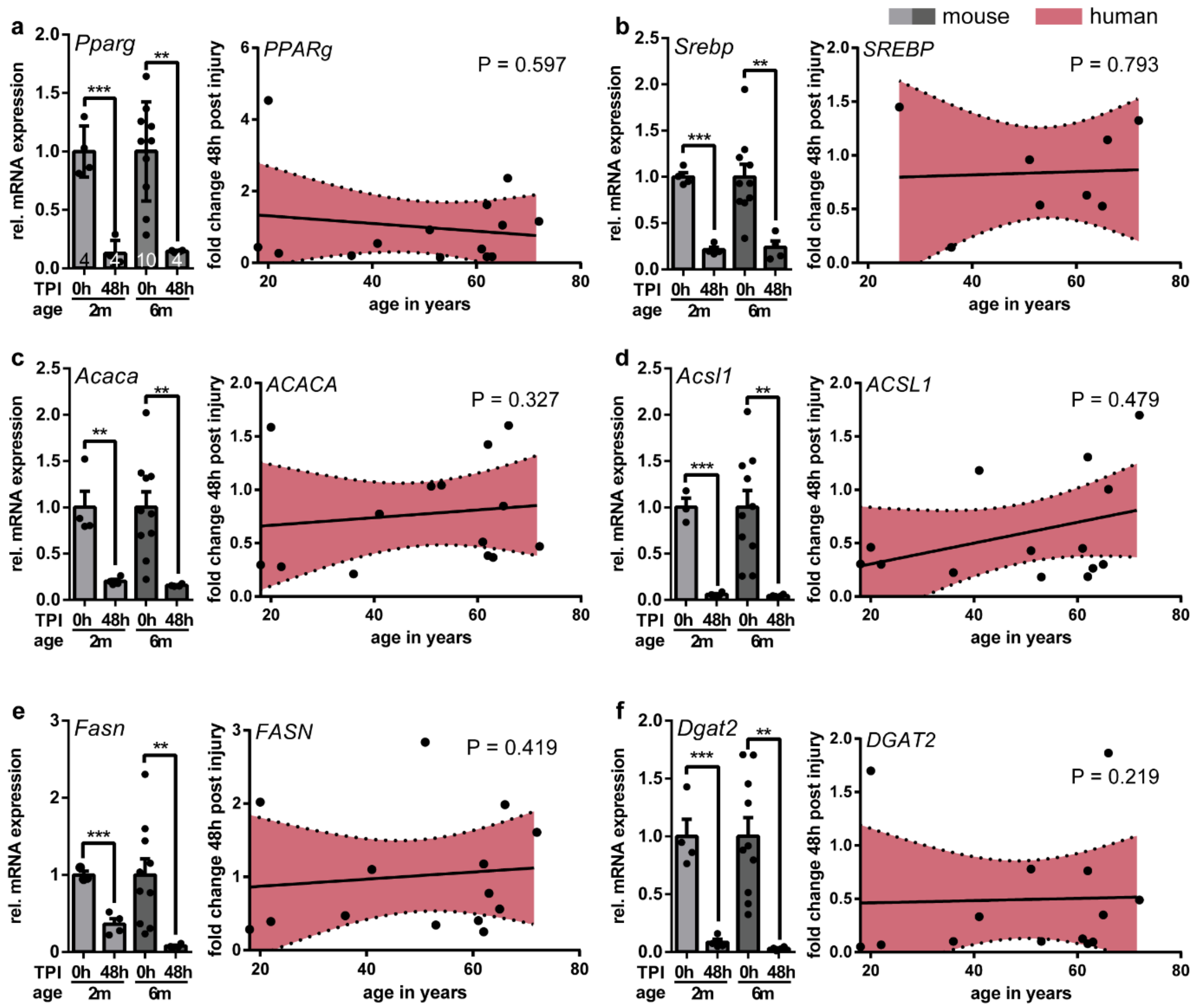

Supplementary Figure 9: Lipogenic gene regulation after injury does not show an age dependency

(**a-f**) qPCR analysis of genes involved in lipid metabolism in sural nerves in the *ex vivo* injury model. Bar charts show gene expression in young (2 months; 2m) vs. old (6 months; 6m) mice, where the gene expression of each 0 h time point was set to 1. Graphs show mean with SEM. Numbers in the graph in (a) indicate the numbers of independent biological replicates analysed for all bar charts (n = 4 for 0 h and 48 h in 2 months old mice, n = 10 and 4 for 0 h and 48 h in 6 months old mice. Two-sided Mann-Whitney test was used to calculate statistical significance in the bar charts (*P < 0.05, **P < 0.005, ***P < 0.001). The scatter plots show gene expression in human nerves 48 h after injury in correlation to patient's age. For each patient 0 h time point was set to 1 and the fold change was calculated for the 48 h time point. Scatter plots depict linear trend line (black line) with the 95% confidence limits (red area). Each dot represents an independent biological replicate (n = 14 for scatter plots in (a), (c), (d), (e), (f) and n = 8 for scatter plot in (b). P values were calculated using the two-sided non-parametrical Spearman correlation. Source data are provided as a Source Data file.

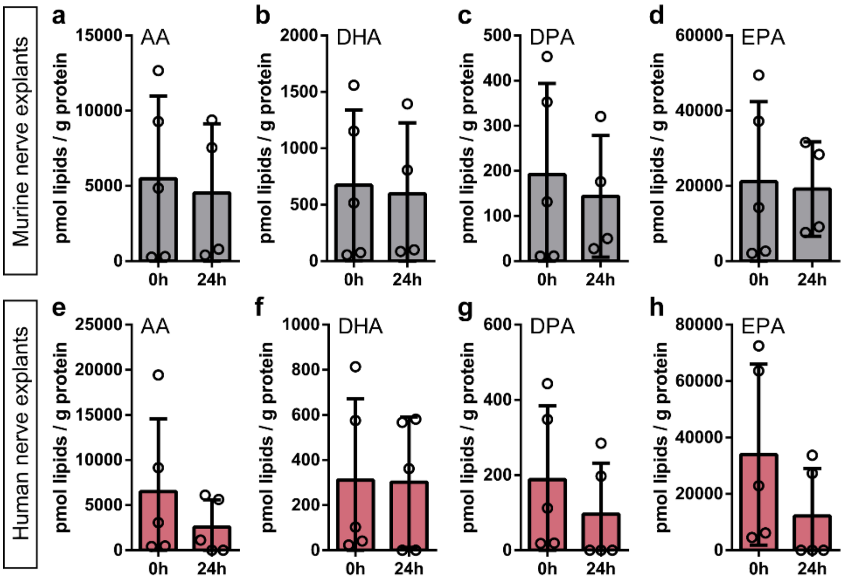

Supplementary Figure 10: Abundance of selected poly unsaturated fatty acids (PUFAs) in injured nerve explants

(**a-h**) Lipidomic analysis of selected PUFAs in control and injured murine (a-d) and human (e-h) sural nerve explants 24 h after injury. Each dot represents a single biological replicate analysed (for mice n = 5 at 0 h and n = 4 at 24 h, for humans n = 5 for 0 h and 24 h). Bars show mean with SD. Source data are provided as a Source Data file.

Abbreviations: AA (arachidonic acid), DHA (docosahexaenoic acid), DPA (docosapentaenoic acid), EPA (eicosapentaenoic acid).

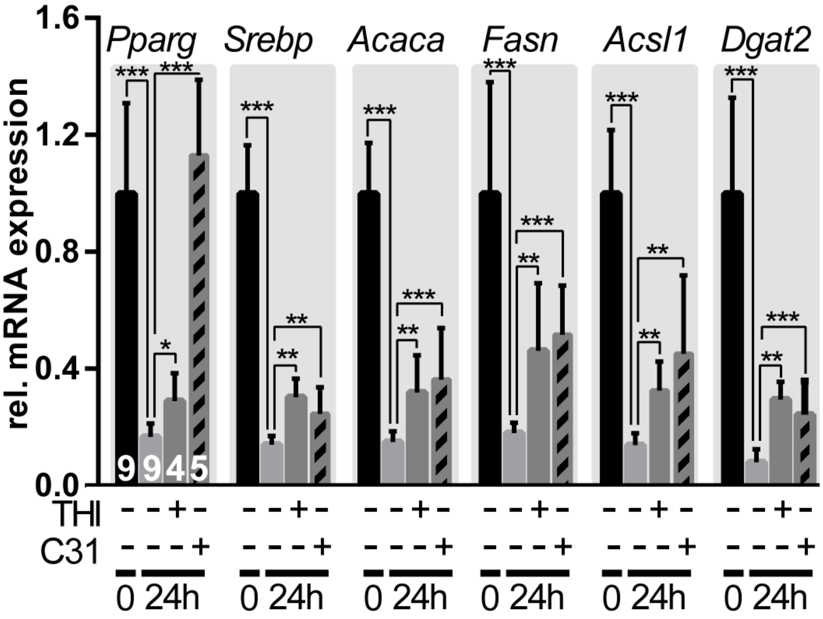

Supplementary Figure 11: S1P dependent regulation of lipogenic genes murine nerves
qPCR analysis of genes involved in lipid metabolism in control or injured murine nerves treated with the SGPL1 inhibitors 2-acetyl-5-tetrahydroxybutyl imidazole (THI) or compound 31 (C31). Expression at 0 h was set to one and the fold change was calculated for the other time points. Numbers in bars indicate independent biological replicates analysed (n = 9, 9, 4, 5 for 0 h, 24 h, 24 h + THI and 24 h + C31 respectively). Bars show mean with SD. Two sided Mann-Whitney test was used to calculate statistical significance (*P < 0.05, **P < 0.005, ***P < 0.001). Source data for are provided as a Source Data file.

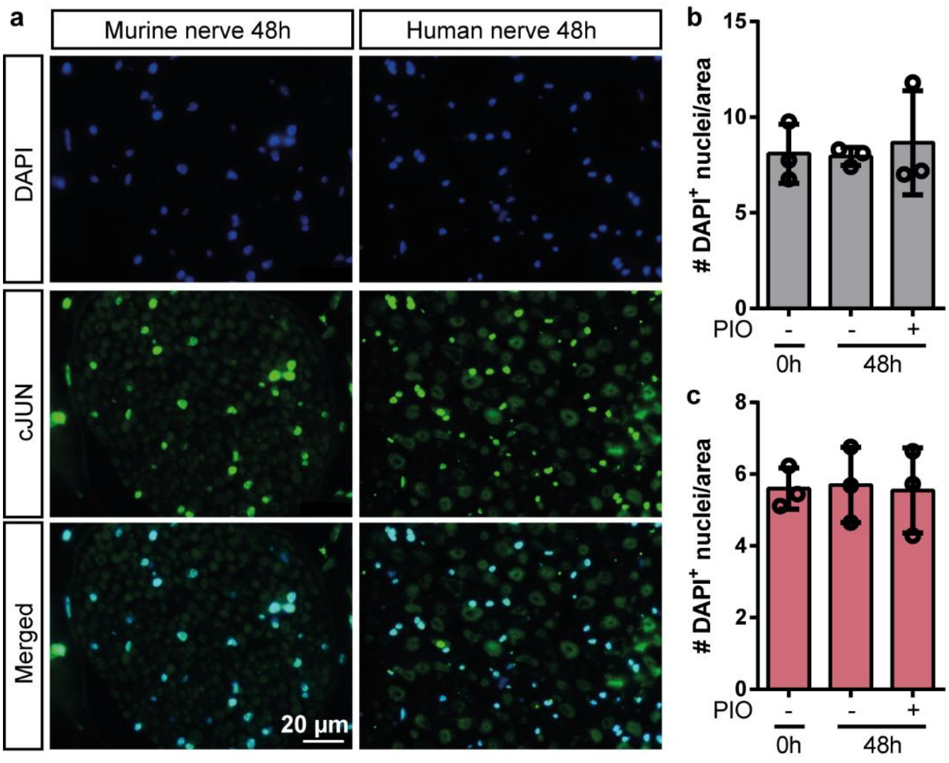

Supplementary Figure 12: Nuclear expression of cJUN in injured murine and human nerves

(**a**) Representative images of murine (left side) and human (right side) control treated injured nerves 48 h post injury. cJUN (green) expression localizes to DAPI^{+} nuclei (blue). Staining was performed in three independent biological replicates for murine and human tissue with similar results.

(**b, c**) Quantification of DAPI^{+} nuclei numbers in murine (b) and human (c) nerves without injury (0 h) or 48 h post injury with control or PIO treatment. Each dot represents one murine or human sample analysed (n = 3 biological replicates for each condition for murine and human tissue). Bars show mean with SD. Source data for are provided as a Source Data file.

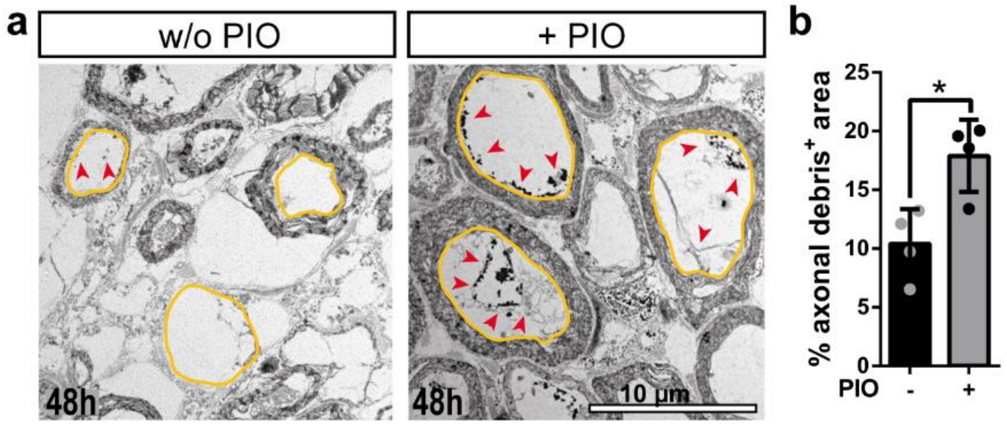

Supplementary Figure 13: Decreased axonal clearance in PIO treated injured nerve explants

(**a**) Electron microscopical pictures of murine nerve explants 48 h upon injury with control (left side) or PIO treatment (right side). Yellow circles mark the area of each axonal compartment. Red arrowheads point at debris inside the axonal compartment. Experiment was performed in four independent biological replicates with similar results.

(**b**) Quantification of the debris covered area of each axonal compartment in %. Each dot represents a single nerve analysed (n = 4 independent biological replicates for each condition). Bars show mean with SD. Two-sided T-test was used to calculate statistical significance (*P < 0.05, **P < 0.005, ***P < 0.001). P = 0.0286. Source data are provided as a Source Data file.

Supplementary Dataset 1.

List of all differentially regulated genes identified by microarray analysis

Differentially regulated genes in murine nerves 2 h and murine and human nerves 24 h upon injury compared to uninjured nerves. Long non-coding RNAs are marked in red.

(separate excel File)

Supplementary methods

Patient cohort used in this study

Supplementary Table 1: List of patients included in the study

The list of patients indicates the sex and age of each patient as well as the analysis in which they were included: HIST (histology), PCR (qPCR), EM (electron microscopy), MA (microarray), LIP (lipidomics), PIO/GW/SR (pioglitazone/GW9662/SR16832 treatment).

Patient	Sex	Age	HIST	PCR	EM	MA	LIP	PIO	GW	SR
**P1**	f	25		+						
**P2**	f	61	+							
**P3**	m	52	+	+						
**P4**	f	43	+							
**P5**	f	71	+							
**P6**	m	50	+							
**P7**	f	65	+	+						
**P8**	f	57	+	+		+				
**P9**	m	36	+	+						
**P10**	m	53	+	+						
**P11**	m	62	+	+		+				
**P12**	m	51	+	+						
**P13**	f	62	+	+						
**P14**	f	66	+	+			+			
**P15**	f	65	+	+						
**P16**	f	20	+	+		+	+			
**P17**	m	26	+	+						
**P18**	m	61	+	+		+				
**P19**	m	51	+	+		+	+			
**P20**	m	67	+	+						
**P21**	m	72	+	+						
**P22**	m	41	+	+			+			
**P23**	m	63	+	+						
**P24**	m	61		+						
**P25**	f	61	+							
**P26**	m	45	+							
**P27**	m	57	+							
**P28**	m	31			+					
**P29**	m	28			+					
**P30**	m	21		+	+				+	
**P31**	f	18		+	+				+	
**P32**	m	22		+					+	
**P33**	f	30						+		
**P34**	f	44						+		
**P35**	f	56						+		
**P36**	f	24		+						
**P37**	m	61					+	+		
**P38**	m	48								+
**P39**	f	52								+
**P40**	f	55								+
**mean age**		**48**								
**median age**		**52**								

TUNEL assay

For the TUNEL assay tissue was fixed in 4% FA followed by preparation of 5 µm paraffin microtome slices. TUNEL assay was performed using the *In Situ* Cell Death Detection Kit (11684795910, Roche) according to the manufacturers protocol. As a positive control for the TUNEL assay (Supplementary Figure 1) we used sections of the cortex of a mouse that received a traumatic brain injury (part of an independent project). Experiments were approved by the local governmental authority for animal experimentation (Regierungspräsidium Tübingen, Germany).

Supplementary material for histology

Primary antibodies: anti-SOX10 (mouse, 1:500, ab212843, Abcam), anti-Histone H3 phospho (rabbit, 1:1000, ab177218, Abcam), anti-CD45 (rat, 1:100, 550539, BD Pharmingen). Secondary antibodies: Alexa 488 (1:500; anti-mouse 488, A-11001, Thermo Fisher Scientific), biotin conjugated secondary antibody anti-rat (1:500; BA-4001, Vectorlabs, Germany). Positive controls for P-HistH3 and CD45 staining (Supplementary Figure 1d, g) are sections of an *in vivo* injured facial nerve, from an independent project, where facial nerve regeneration is addressed. Experiments were approved by the local governmental authority for animal experimentation (Regierungspräsidium Tübingen, Germany).

Murine nerve treatment with SGPL1 inhibitors

Murine nerves were treated with 2-acetyl-5-tetrahydroxybutyl imidazole (THI; Sigma-Aldrich; dissolved in DMSO; final concentration 100µM) or compound 31 (C31; Glixxlabs; dissolved in

DMSO; final concentration 5 µM). Treatment was performed by bath application in Ringer solution plus each substance, starting immediately after dissection.

List of primers used in the study

Supplementary Table 2: Primer pairs used for qPCR analysis of murine (m) and human (h) samples

Primer	Fwd sequence (5′ > 3′)	Rev sequence (5′ > 3′)
cFos m	CCT GCC CCT TCT CAA CGA C	GCT CCA CGT TGC TGA TGC T
cFos h	GGG GCA AGG TGG AAC AGT TA	AGT TGG TCT GTC TCC GCT TG
Egr2 m	GTT GAC TGT CAC TCC AAG AAA TGG	AGC GCA GCC CTG TAG GC
Egr2 h	AGC TTT GCT CCC GTC TCT G	AGC TGG CAC CAG GGT ACT
cJun m	ACC CCC ACT CAG TTC TTG TG	AGT TGC TGA GGT TGG CGT AG
cJun h	GCA AAG ATG GAA ACG ACC TTC T	GCT CTC GGA CGG GAG GAA
Brn2 m/h	GAA AAG GAT GAC CCC TCC CG	TGT GGT GGA GTG TCC CTA CT
Atf3 m	GCT GGA GTC AGT TAC CGT CAA	CGC CTC CTT TTC CTC TCA T
Atf3 h	GCT GTC ACC ACG TGC AGT ATC TC	CTG TTC CTC CTC TTG CTG ACA AGC
Pmp22 m	ATC TCA AAG CCT TCG TCA CTC C	GGC CAA TAC AAG TCA TCG CTA G
Pmp22 h	ATC ATA AAG CCT TCA TCA CTC C	GGC CAA TAC AAG TCA TTG CCA G
Gdnf m/h	GAG AGG AAT CGG CAG GCT GCA GCT G	CAG ATA CAT CCA CAC CGT TTA GCG G
Erbb2 m	GCA AGC ACT GTC TGC CAT GC	GGG CAC AAG CCT CAC ACT GG
Erbb2 h	CCT CTG ACG TCC ATC ATC TC	ATC TTC TGC TGC CGT CGC TT
Shh m	TTA AAT GCC TTG GCC ATC TC	CCA CGG AGT TCT CTG CTT TC
Shh h	TCC AGA AAC TCC GAG CGA TTT AAG	TCC TGG CCA CTG GTT CA
Pparg m/h	GAA AGA CAA CGG ACA AAT CAC C	GGG GGT GAT ATG TTT GAA CTT G
Srebp m	CGG TCT TTC TAT CAA TGA CA	TGC GCA AGA CAG CAG ATT TA
Srebp h	CGC TCC TCC ATC AAT GAC A	TGC GCA AGA CAG CAG ATT TA
Acaca m	GCG TCG GGT AGA TCC AGT T	CTC AGT GGG GCT TAG CTC TG
Acaca h	GCG TCG GGT GGA CCC AGT C	CTC AGC TGT GCT TAG CTC TG
Acsl1 m	ATC TGG TGG AAC GAG GCA AG	TCC TTT GGG GTT GCC TGT AG
Acsl1 h	AAC TGG TGG AAC GAG GCC AG	TCC TTT GGG GTT GCC TGT AG
Fasn m	GCT GCT GTT GGA AGT CAG C	AGT GTT CGT TCC TCG GAG TG
Fasn h	GCT GCT GCT GGA AGT CAC C	AGT GTG TGT TCC TCG GAG TG
Dgat2 m	GCT GGT GCC CTA CTC CAA G	CCA GCT TGG GGA CAG TGA
Dgat2 h	GCT GGT GCC CTA CTC CAA G	CCA GCT TGG GGA TGG TGA
Ehhadh m	CTA GCC CAG AGC ACA TTG A	GCC CAA CTG AGG CAG CAT AGT AC
Ehhadh h	CTA GCC CAG AGC ACA TTG A	ACC CAA CTG TGG AAG CAT AGA AC
Echs1 m	CAA CCA AGC ACT GGA GAC CT	TGC ATT TCC TTG ATG TCA GC
Echs1 h	TGG AGA TGG TCC TCA CTG GT	CCA CCA GTG TCT CAA CAG GA
Medag m	GTG CCA ATC AAC CAG TGA CA	GCC CTG ATG TCC AGT GTA CC
Medag h	AGG ACG TAC GCG TTT CTT GT	GAA TTA CTG AGC CCG AAC CA
Mbp m	ACT TCC AGG CCA GCT ATC CC	CCC TTT CCT TCC CTC TGC C
Rel m	ACA ACA ACC GGA CAT ACC CG	GGT CTG CGT TCT GGT CCA A

Primer	Fwd sequence (5′ > 3′)	Rev sequence (5′ > 3′)
Rel h	TGA ACA TGG TAA TTT GAC GAC TG	ACA CGA CAA ATC CTT AAT TCT GC
Ccl2 m	CCC AAT GAG TAG GCT GGA GA	TCT GGA CCC ATT CCT TCT TG
Ccl2 h	AGT CTC TGC CGC CCT TCT	GTG ACT GGG GCA TTG ATT G
Ccl7 m	GCT GCT TTC AGC ATC CAA GTG	GCA GCA TGT GGA TGC ATT G
Ccl7 h	GAA AGC CTC TGC AGC ACT TC	AAT CTG TAG CAG CAG GTA GTT GAA
Cxcl10 m	GCT GCC GTC ATT TTC TGC	TCT CAC TGG CCC GTC ATC
Cxcl10 h	GAA AGC AGT TAG CAA GGA AAG GT	GAC ATA TAC TCC ATG TAG GGA AGT GA
Il-1β m	TGT AAT GAA AGA CGG CAC ACC	TCT TCT TTG GGT ATT GCT TGG
Il-1β h	ACA GAT GAA GTG CTC CTT CCA	GTC GGA GAT TCG TAG CTG GAT
Ptgs2 m	TGC CTC CCA CTC CAG ACT AGA	CAG CTC AGT TGA ACG CCT TTT
Ptgs2 h	CTT CAC GCA TCA GTT TTT CAA G	TCA CCG TAA ATA TGA TTT AAG TCC AC

Functional annotation cluster analysis

Functional annotation cluster analysis was performed using the functional annotation cluster clustering tool of the online DAVID platform (DAVID Bioinformatics Resources 6.8, NIAID/NIH).
