# Supplementary material for: Lipid metabolism adaptations are reduced in human compared to murine Schwann cells following injury
Source: Nat Commun. 2020 May 1;11:2123. doi: 10.1038/s41467-020-15915-4 (PMC7195462; doi:10.1038/s41467-020-15915-4)
Supplement: Supplementary file 3 — Description of Additional Supplementary Files [file 41467_2020_15915_MOESM3_ESM.docx]

Description of Additional Supplementary Files

**Title: Supplementary Dataset 1: List of all differentially regulated genes identified by microarray analysis**

**Description:** Differentially regulated genes in murine nerves 2 h and murine and human nerves 24 h upon injury compared to uninjured nerves. Long non-coding RNAs are marked in red
