# Supplementary material for: Lipid metabolism adaptations are reduced in human compared to murine Schwann cells following injury
Source: Nat Commun. 2020 May 1;11:2123. doi: 10.1038/s41467-020-15915-4 (PMC7195462; doi:10.1038/s41467-020-15915-4)
Supplement: Supplementary file 5 — Reporting Summary [file 41467_2020_15915_MOESM5_ESM.pdf]

nature research

Corresponding author(s): Sofia Meyer zu Reckendorf (sofia.meyer-zu-reckendorf@uni-ulm.de)
Bernd Knöll (bernd.knoell@uni-ulm.de)

Last updated by author(s): Mar 29, 2020

Reporting Summary

Nature Research wishes to improve the reproducibility of the work that we publish. This form provides structure for consistency and transparency in reporting. For further information on Nature Research policies, see Authors & Referees and the Editorial Policy Checklist .

Statistics

For all statistical analyses, confirm that the following items are present in the figure legend, table legend, main text, or Methods section.

n/a	Confirmed	
☐	☒	The exact sample size (*n*) for each experimental group/condition, given as a discrete number and unit of measurement
☐	☒	A statement on whether measurements were taken from distinct samples or whether the same sample was measured repeatedly
☐	☒	The statistical test(s) used AND whether they are one- or two-sided<br>*Only common tests should be described solely by name; describe more complex techniques in the Methods section.*
☐	☒	A description of all covariates tested
☒	☐	A description of any assumptions or corrections, such as tests of normality and adjustment for multiple comparisons
☐	☒	A full description of the statistical parameters including central tendency (e.g. means) or other basic estimates (e.g. regression coefficient) AND variation (e.g. standard deviation) or associated estimates of uncertainty (e.g. confidence intervals)
☐	☒	For null hypothesis testing, the test statistic (e.g. *F*, *t*, *r*) with confidence intervals, effect sizes, degrees of freedom and *P* value noted<br>*Give P values as exact values whenever suitable.*
☒	☐	For Bayesian analysis, information on the choice of priors and Markov chain Monte Carlo settings
☒	☐	For hierarchical and complex designs, identification of the appropriate level for tests and full reporting of outcomes
☒	☐	Estimates of effect sizes (e.g. Cohen's *d*, Pearson's *r*), indicating how they were calculated

Our web collection on statistics for biologists contains articles on many of the points above.

Software and code

Policy information about availability of computer code

Data collection	Histology image acquisition: AxioVision 4.9 (Zeiss)<br>qPCR data acquisition: LightCycler® 480 II Software (Roche)
Data analysis	Histological analysis: ImageJ2<br>Microarray data analysis: Affymetrix® Expression Console™, BRB-ArrayTools v4.6.0, GO Miner (version from 2017-01), Pscan Ver. 1.5<br>Lipidomic analysis: MetaboAnalyst 4.0, Multiquant 3.0.3<br>Statistical analysis: GraphPad Prism 6.01<br>Functional annotation cluster analysis: DAVID Bioinformatics Resources 6.8, NIAID/NIH

For manuscripts utilizing custom algorithms or software that are central to the research but not yet described in published literature, software must be made available to editors/reviewers. We strongly encourage code deposition in a community repository (e.g. GitHub). See the Nature Research guidelines for submitting code & software for further information.

Data

Policy information about availability of data

All manuscripts must include a data availability statement. This statement should provide the following information, where applicable:

- Accession codes, unique identifiers, or web links for publicly available datasets
- A list of figures that have associated raw data
- A description of any restrictions on data availability

All data generated for this publication are included in this article and its supplementary materials. Any additional information/data are available from the corresponding authors upon reasonable request.

Field-specific reporting

Please select the one below that is the best fit for your research. If you are not sure, read the appropriate sections before making your selection.

- [x] Life sciences
- [ ] Behavioural & social sciences
- [ ] Ecological, evolutionary & environmental sciences

For a reference copy of the document with all sections, see nature.com/documents/nr-reporting-summary-flat.pdf

Life sciences study design

All studies must disclose on these points even when the disclosure is negative.

Sample size	For analysis of human tissue the maximal sample size possible was used for each application (max. possible size was 40 individuals due to the ethical approval). Due to the limited amount of material it was not possible to perform all analyzes with all patients. For analysis on murine tissue a sample size between 3-10 was chosen. The minimum sample size of 3 war chosen in order to be able to perform statistics. The maximum size of up to 10 was chosen for sensitive methods (e.g. RNA isolation, qPCR) in order optain enough samples and limit variation. Due to the smal size of murine sural nerves, material was very limited and for some replicates not enough to perform all experiments. In addition, samples had to be discarted when due to the limited amount the quality was not sufficient (e.g. RNA degraded or too low amount). In some cases, single samples were excluded if detected as outliers (see next point).
Data exclusions	Outliers were identified (and excluded) using the ROUT function of the GraphPad Prism 6 software.
Replication	For human material: RNA isolation, qPCR, histology and electron microscopy were performed individually for each patient. For lipidomic analysis the lipid extraction and analysis were performed at the same time for all 5 patients (5 biological replicates). PIO, SR and GW treatment and subsequent RNA isolation was performed for each patient separately.<br>For murine tissue: RNA isolation, qPCR and histology were performed in at least 3 independent experiments each including several biological replicates. Tissue for electron microscopy was prepared at the same time for all 4 mice (4 biological replicates). The same is the case for teased fibers (4 biological replicates prepared and processed at the same time), pioglitazone treatment (4 biological replicates prepared and processed at the same time) and lipid extraction and lipidomic analysis (5 biological replicates prepared and processed at the same time). In case of DOP (4-deoxypyridoxine) treatment, two independent experiments were performed with 5 and 4 biological replicates each.<br>In all cases all replication attemts were succsesfull and all data were included in the analyses.
Randomization	Not relevant. Each patient sample was divided and used for all time points needed. Which patient was used for which type of analysis was assigned arbitrarily according to the current experiments performed when sample was received. For murine samples we used C57BL/6J mice of either 2 or 6 months of age (as indicated). No randomization was necessary since all mice of one age are practically identical.
Blinding	No blinding was necessary. All analyses were performed using quantifiable parameters (e.g. Ct Values for qPCR, thresholds for histology etc.) so no bias was possible.

Reporting for specific materials, systems and methods

We require information from authors about some types of materials, experimental systems and methods used in many studies. Here, indicate whether each material, system or method listed is relevant to your study. If you are not sure if a list item applies to your research, read the appropriate section before selecting a response.

Materials & experimental systems

n/a	Involved in the study	
[ ]	[x]	Antibodies
[x]	[ ]	Eukaryotic cell lines
[x]	[ ]	Palaeontology
[ ]	[x]	Animals and other organisms
[ ]	[x]	Human research participants
[x]	[ ]	Clinical data

Methods

n/a	Involved in the study	
[x]	[ ]	ChIP-seq
[x]	[ ]	Flow cytometry
[x]	[ ]	MRI-based neuroimaging

Antibodies

Antibodies used	Primary Antibodies:<br>Anti-neuronal class III beta-Tubulin monoclonal antibody [TUJ1], BioLegend MMS-435P, lot: B24-9869<br>Anti-S100 beta recombinant antibody [EP1576Y], abcam, ab52642, lot: GR321 5095-1<br>Anti-PPAR gamma polyclonal antibody, abcam, ab45036, lot: GR319 2046-5<br>Anti-cJUN monoclonal antibody [60A8], Cell Signaling, #9165, lot: 11<br>Anti-Histone H3 (phospho S10) recombinant antibody [EPR17246], abcam, ab177218, lot: GR208 177-2<br>Anti-Mouse CD45 antibody [30-F11], BD Pharmingen, 550539, lot: 6021713<br>Anti-SOX10 mouse monoclonal antibody [SOX10/991], abcam, ab212843, lot: GR3225 224-2

Sekondary antibodies:
Biotin conjugated secondary antibody anti-rabbit, Vectorlabs, BA-1000, lot: ZE0730
Biotin conjugated secondary antibody anti-rat, Vectorlabs, BA-4001, lot: Y0809
Alexa488 anti-rabbit, Thermo Fisher Scientific, A-11008, lot: 1981125
Alexa546 anti-mouse, Thermo Fisher Scientific, A-11003, lot: 2012506
Alexa 488 anti-mouse, Thermo Fisher Scientific, A-11001, lot: 1726530

Validation

All antibodies are commercially available, have been validated according to the manufacturer's website and are frequently used in publications. We regularly test new antibodies by using negative controls during staining (using only secondary antibody or using tissue that should be negative for the specific protein). In addition, we use positive controls if necessary (e.g. CD45 and P-Histone H3 antibodies in Figure S1 in this study).
Valiadation according to manufacturers website:

Anti-neuronal class III beta-Tubulin monoclonal antibody [TUJ1], BioLegend MMS-435P, lot: B24-9869:
Each lot of this antibody is quality control tested by formalin-fixed paraffin-embedded immunohistochemical staining. This antibody is well characterized and highly reactive to neuron specific Class III β-tubulin (βIII). TUJ1 does not identify β-tubulin found in glial cells. TUJ1 recognizes an epitope located within the last 15 C-terminal residues (Lee MK, et al. 1990. Proc. Natl. Acad. Sci. USA 18:7195.). Further publications:
Jongbloets J, et al. 2017. Nat Commun. 8: 14666.
Liu W.J, et al. 2015. Eur J Histochem. 59(1): 2464.
Ambasudhan R, et al. 2011. Cell Stem Cell. 9(2):113.
Hu X., et al. 2006. Nature Neuroscicene. 9(12):1520.
Zechner D., et al. 2003. Develop Biology. 258(2):406.

Anti-S100 beta recombinant antibody [EP1576Y], abcam, ab52642, lot: GR321 5095-1:
This product is a recombinant monoclonal antibody, which offers several advantages including:
- High batch-to-batch consistency and reproducibility
- Improved sensitivity and specificity
- Long-term security of supply
- Animal-free production
Positive controls for IHC: Human, mouse and rat cerebral cortex. Human spiral ganglion and melanoma tissue; Normal WT and laser-treated mouse retina; Native and acellular peripheral nerve sections; Embryonic mouse brain tissue, brain tissue.
Our RabMAb® technology is a patented hybridoma-based technology for making rabbit monoclonal antibodies.
Publications:
Wanner R. et al. 2019, Front Neurol. 10:310.
Carvalho TG. et al. 2019, Neurochem Int. 126:218-228.

Anti-PPAR gamma polyclonal antibody, abcam, ab45036, lot: GR319 2046-5:
Specificity: Ab45036 detects peroxisome proliferator activated receptor (PPAR) gamma 2. This antibody does not detect PPAR alpha or PPAR delta. This sequence is from P37231-1 (Isoform 2), the sequence is not present in P37231-2 (Isoform 1) or P37231-3 (Isoform 3).
Positive control: NIH-3T3 cell lysate
Publications:
Zheng F. and Cai Y. 2019, Lipids Health Dis. 18(1):6.
Desmarais F. et al. 2019, Biochim Biophys Acta Mol Cell Biol Lipids. 1864(4):522-531

Anti-cJUN monoclonal antibody [60A8], Cell Signaling, #9165, lot: 11:
This antibody has been validated using SimpleChIP® Enzymatic Chromatin IP Kits.
Specificity / Sensitivity: c-Jun (60A8) Rabbit mAb detects endogenous levels of total c-Jun protein, regardless of phosphorylation state.
Source / Purification: Monoclonal antibody is produced by immunizing animals with a GST-c-Jun protein corresponding to the amino-terminal sequence of human c-Jun.
Publications:
Pang D. et al. 2020, Biol Open. 9(2)
Wang W. et al. 2020, Cell Death Dis. 11(2):107

Anti-Histone H3 (phospho S10) recombinant antibody [EPR17246], abcam, ab177218, lot: GR208 177-2:
This product is a recombinant monoclonal antibody, which offers several advantages including:
- High batch-to-batch consistency and reproducibility
- Improved sensitivity and specificity
- Long-term security of supply
- Animal-free production
Positive control IHC-P: Human tonsil; Human ovarian carcinoma; Mouse spleen; Rat spleen.
Our RabMAb® technology is a patented hybridoma-based technology for making rabbit monoclonal antibodies. For details on our patents, please refer to RabMAb® patents.

Publications:
Jia S. et al. 2019, Cell Death Dis. 10(2):142
Curt J.R. 2019, Elife. 8.

Anti-Mouse CD45 antibody [30-F11], BD Pharmingen, 550539, lot: 6021713:
The 30-F11 clone has been reported to react with all isoforms and both alloantigens of CD45, which is found on hematopoietic stem cells and all cells of hematopoietic origin, except erythrocytes.
No further information regarding validation. This antibody has been validated in our laboratory using positive controls (in vivo injured peripheral nerve with infiltration of peripheral immune cells) and negative controls (uninjured nerves lacking immune cells).

Anti-SOX10 mouse monoclonal antibody [SOX10/991], abcam, ab212843, lot: GR3225 224-2 :
Positive control IHC-P: Human melanoma and mouse brain tissue.
Publications:
Liu L.P. et al., 2019, Cell Rep. 27(2):455-466.
Corces M.R- et al., 2017, Nat Methods. 14(10):959-962.

Animals and other organisms

Policy information about studies involving animals; ARRIVE guidelines recommended for reporting animal research

Laboratory animals	C57BL/6J mice, age 2 months, both sexes (bred in house)<br>C57BL/6J mice, age 6 months, male (acquired from Janvier Labs)
Wild animals	No wild animals were used in this study.
Field-collected samples	No field-collected samples were used in this study.
Ethics oversight	No experiments were performed on living animals. Approval for laboratory animal killing and tissue dissection as performed in this study was granted by the Regierungspräsidium Tübingen, Germany (local governmental authority for animal experimentation).

Note that full information on the approval of the study protocol must also be provided in the manuscript.

Human research participants

Policy information about studies involving human research participants

Population characteristics	List of patients (listed also in Supplementary Table 1)<br>Patient/ Sex (female or male)/ Age (years):<br>P1 /f /25<br>P2 /f /61<br>P3 /m /52<br>P4 /f /43<br>P5 /f /71<br>P6 /m /50<br>P7 /f /65<br>P8 /f / 57<br>P9 /m /36<br>P10 /m /53<br>P11 /m /62<br>P12 /m / 51<br>P13 /f /62<br>P14 /f /66<br>P15 /f /65<br>P16 /f /20<br>P17 /m / 26<br>P18 /m /61<br>P19 /m /51<br>P20 /m /67<br>P21 /m /72<br>P22 /m /41<br>P23 /m /63<br>P24 /m /61<br>P25 /f /61<br>P26/ m/ 45<br>P27/ m/ 57<br>P28/ m/ 31

	P29/ m/ 28<br>P30/ m/ 21<br>P31/ f/ 18<br>P32/ m/ 22<br>P33/ f/ 30<br>P34/ f/ 44<br>P35/ f/ 56<br>P36/ f/ 24<br>P37/ m/ 61<br>P38/ m/ 48<br>P39/ f/ 52<br>P40/ f/ 55<br><br>All patients had a primary peripheral nerve injury of the limbs or plexus, which did not include the sural nerve. Patients underwent nerve reconstruction surgery during which the sural nerve was dissected and used as auto-transplant. Not used parts of the sural nerves were used for experients during this project.
Recruitment	Patients were informed prior to surgery about the possibility to donate tissue for this project and had to give their written informed consent in order to be included. Only patients with a minimum age of 18 years were included. All patients that where operated by our cooperations partners in the Neurosurgery Clinic in Günzburg throughout the duration of the project where informed about the possibility to donate their tissue. Hence, patients where not selected according to any biased criteria. This is reflected by the wide age distribution and approximately equal numbers of female and male patients.
Ethics oversight	All procedures performed using human tissue were approved by the ethics committee of Ulm University.

Note that full information on the approval of the study protocol must also be provided in the manuscript.
